# Effect of Topical Nepafenac on Central Foveal Thickness following Panretinal Photocoagulation in Diabetic Patients

**DOI:** 10.1155/2017/3765253

**Published:** 2017-06-27

**Authors:** Nahla B. Abu Hussein, Ahmed A. Mohalhal, Dalia A. Ghalwash, Ahmed A. Abdel-Kader

**Affiliations:** Faculty of Medicine, Cairo University, Cairo, Egypt

## Abstract

**Purpose:**

To evaluate effectiveness of topical nepafenac in reducing macular edema following panretinal photocoagulation (PRP).

**Design:**

Prospective randomized double-blinded controlled study.

**Methods:**

Sixty eyes of 60 patients having proliferative or severe nonproliferative diabetic retinopathy had PRP. Patients were then divided into two groups: nepafenac group (30 eyes) receiving 1% topical nepafenac eye drops for 6 months and control group (30 eyes) receiving carboxymethylcellulose eye drops for 6 months. Best-corrected visual acuity (BCVA) and macular optical coherence tomography were followed up at 1, 2, 4, and 6 months after PRP.

**Results:**

BCVA was significantly better in nepafenac group than in control group at all follow-ups (*P* < 0.01). At 6 months post-PRP, logMAR BCVA was 0.11 ± 0.04 (equivalent to 20/26 Snellen acuity) in the nepafenac group and 0.18 ± 0.08 (equivalent to 20/30 Snellen acuity) in the control group (*P* < 0.01). Central foveal thickness (CFT) increased in both groups from the first month after PRP. Increase in CFT was higher in control group than in nepafenac group throughout follow-up, but the difference became statistically significant only after 4 months. No significant ocular adverse events were reported with topical nepafenac.

**Conclusion:**

Topical nepafenac can minimize macular edema and stabilize visual acuity following PRP for diabetic patients.

## 1. Introduction

Topical nonsteroidal anti-inflammatory drugs (NSAIDs) have been used to treat allergic conjunctivitis, prevent intraoperative miosis during intraocular surgery, decrease postoperative pain and inflammation, and treat pseudophakic macular edema [1].

There is growing evidence that an inflammatory mechanism has a role in the pathogenesis of diabetic retinopathy [2]. Breakdown of blood retinal barrier and chronic subclinical inflammation are evident in cases with diabetic macular edema [3]. Animal and human studies have shown an increased level of inflammatory mediators and prostaglandins (PGs) in the vitreous cavity of patients with diabetic retinopathy and that the vitreous level of prostaglandin PGE_2_ is directly related to that of vascular endothelial growth factor (VEGF) [4, 5]. In addition, intravitreal triamcinolone has been shown to reduce the risk of worsening of proliferative diabetic retinopathy (PDR) [6].

Despite the beneficial effect of panretinal photocoagulation (PRP) in reducing moderate visual loss by 50% for cases with high-risk PDR, it can result in macular edema and cause a significant decrease in visual acuity [7]. The macular edema is thought to result from the inflammatory response following the laser treatment [8]. The aim of this study is to evaluate whether the anti-inflammatory effect of nepafenac eye drops can help to stabilize the visual acuity and decrease the risk of progression of macular edema following PRP for diabetic retinopathy.

## 2. Methods

The study was approved by Cairo University research ethics committee and followed the tenets of the Declaration of Helsinki. Patients were recruited from the retina service clinic in Cairo University Hospital during the period from January 2013 through December 2015. An informed consent was obtained from all patients.

In this prospective interventional institutional study, diabetic patients with severe nonproliferative diabetic retinopathy or proliferative diabetic retinopathy scheduled for panretinal photocoagulation and having no diabetic macular edema were enrolled. All patients were treatment naive. Diabetic macular edema was defined as a central foveal thickness of >210 *μ*m or a mean central macular thickness in 3D map of >300 *μ*m.

Patients were excluded from the study if they had media opacity, other ocular diseases, or had a history of prior ocular surgery or laser treatment. In addition, as the study involved long-term use of nepafenac, the risk of corneal problems was considered. Therefore, patients with dry eye syndrome or corneal epithelial problems were also excluded from the study. Patients who developed advanced diabetic disease requiring vitrectomy were excluded from the analysis. We also excluded patients with intolerance to nepafenac drops and those who could not complete the 6 months follow-up.

### 2.1. Sample Size Calculation

An estimation of the sample size was performed considering a study power of at least 0.9 with an alpha error of 0.05 aiming to detect a difference of 15 *μ*m in central foveal thickness (CFT) 6 months after PRP between the 2 groups, assuming a standard deviation of 15 *μ*m. Based on this estimation, a total of 44 eyes were found to be adequate, and the recruitment of at least 60 eyes was targeted.

All patients had a detailed history taking and completed ophthalmic examination, including slit lamp examination, corneal fluorescein staining, intraocular pressure (IOP) measurement, fundus examination, visual acuity measurements using Snellen's acuity chart, fluorescein angiography using TRC 50 DX fundus camera (Topcon Inc., Tokyo, Japan), and spectral domain optical coherence tomography (SD-OCT) using 3D OCT 2000 (Topcon Inc., Tokyo, Japan). Six radial line scans through the centre of the foveal lesions were used to determine the presence of fluid in the macula. The retinal thickness map analysis protocol was used to display numeric averages of the measurements for each of the nine subfields. The mean thickness at the point of intersection of the six radial scans was defined as central foveal thickness (CFT). All visual acuity assessments and macular thickness measurements by OCT were done by one of the authors (NBA) who was masked to the received treatment.

All patients had panretinal photocoagulation treatment completed in 4 sessions over 4 weeks using argon green laser with a retinal spot size of 200–500 *μ*m, a duration of 0.1 second, and an intensity of 200–500 mW until a gray burn spot was evident. Patients received 1500–2000 laser shots (375–500 laser shots/session/eye). Laser treatment was done by two of the authors (AM and AA) who were masked to the postlaser regimen. Patients with new or residual retinal neovascularization during follow-up had further augmentation of laser treatment.

Following laser treatment, patients were randomized using a random table to receive either nepafenac (NEVANAC® ophthalmic suspension 0.1%; Alcon Research Ltd., Fort Worth, TX) eye drops t.i.d. (nepafenac group) or carboxymethylcellulose drops t.i.d. (control group) for 6 months. Both patients and examiners were double blinded about the received drops.

Patients were followed up at 1, 2, 4, and 6 months after laser treatment. Best-corrected visual acuity (BCVA) was measured using Snellen's acuity chart converted to the logarithm of the minimal angle of resolution (logMAR) scale for statistical analysis. Changes in the macular thickness and macular morphology were studied using OCT at each follow-up. Tolerance to the drug and local symptoms as itching, redness, or burning sensation were documented. In addition, patients were monitored for safety outcomes during each follow-up. Safety outcomes included any corneal changes (using slit lamp examination and fluorescein staining) such as corneal edema, punctuate erosions, corneal thinning or ulceration, changes in intraocular pressure, cataract formation or progression, and ocular inflammation.

Comparison between the 2 groups was done using Mann-Whitney test for continuous variables and chi-square test for categorical variables. All statistical calculations were done using SPSS (Statistical Package for the Social Sciences v 24, SPSS Inc., Chicago, IL, USA).

## 3. Results

Sixty eyes of 60 patients were randomized into 2 groups: the nepafenac group (*n* = 30 eyes) and the control group (*n* = 30 eyes). The mean age of the studied patients was 46.9 ± 6.1 years. There were no significant differences between the baseline characteristics of the studied patients of both groups (Table 1). Fifteen eyes (25%) needed additional PRP due to residual or new retinal neovascularization: 7 eyes in group A and 8 eyes in group B.

### 3.1. Visual Acuity

**Figure 1:**
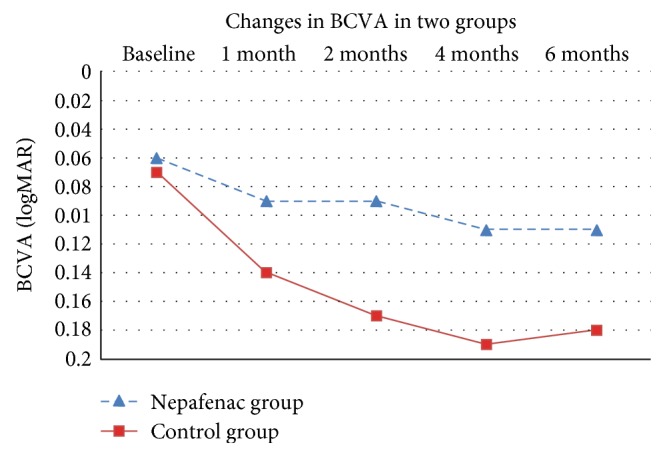
Best-corrected visual acuity (BCVA) at baseline and during follow-ups in nepafenac and control groups.

The mean BCVA before PRP was 0.06 ± 0.06 logMAR in the nepafenac group and 0.07 ± 0.07 logMAR in the control group (Figure 1 and Table 2). The difference was statistically insignificant (*P* = 0.40). Following laser treatment, there was worsening of BCVA in both groups. The mean number of lost lines at 6 months post-PRP was significantly higher (*P* < 0.01) in the control group (1.96 ± 1.07) than in the nepafenac group (1.0 ± 1.0).

The BCVA was significantly better in the nepafenac group compared to that in the control group at all follow-up periods (*P* ≤ 0.01). At 6 months post-PRP, the BCVA was 0.11 ± 0.04 (equivalent to 20/26 Snellen acuity) in the nepafenac group and 0.18 ± 0.08 (equivalent to 20/30 Snellen acuity) in the control group (*P* = 0.007).

### 3.2. Central Foveal Thickness

**Figure 2:**
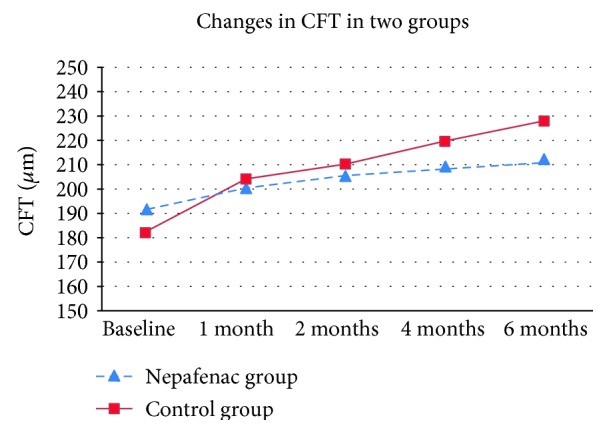
Central foveal thickness (CFT) at baseline and during follow-ups in nepafenac and control groups.

The mean CFT before laser treatment was 191.63 *μ*m ± 8.82 in the nepafenac group and 182.68 *μ*m ± 9.55 in the control group. The difference was statistically insignificant (*P* = 0.41). Following laser treatment, there was an increase in the CFT in both groups. The increase in CFT was higher in the control group than in the nepafenac group starting from the first month after PRP but became statistically significant only at 4 and 6 months after PRP (Figure 2 and Table 3). At 6 months post-PRP, the CFT was 210 *μ*m ± 0.11 in the nepafenac group and 228 *μ*m ± 20 in the control group (*P* = 0.002). None of the patients showed changes in the macular morphology following laser treatment.

### 3.3. Safety Outcomes

Ocular adverse events reported during the follow-up are listed in Table 4. None of the patients developed punctuate epithelial keratopathy or corneal changes during follow-up. There was no significant difference in the mean IOP at 6 months between the treatment group (16.6 ± 2.1 mmHg) and the control group (16.4 ± 2.2 mmHg).

## 4. Discussion

Nepafenac is a topical NSAID that is made in a prodrug form. It has rapid corneal penetration ability. In an in vitro study, it showed six folds greater corneal penetration than diclofenac [9]. After corneal penetration, it gets deaminated to form the active metabolite, amfenac. Activation ensues by hydrolases within the uveal tissue and retina. Because activation is targeted to the uveal tissue, nepafenac may have prolonged activity in these highly vascular tissues of the eye [10, 11]. Nepafenac and its active metabolite amfenac are potent inhibitors of the cyclooxygenase enzyme isoforms (COX1 and COX2) [12]. Animal and human studies showed increased levels of inflammatory mediators and prostaglandins (PGs) in the vitreous cavity in diabetic retinopathy due to breakdown of the blood retinal barrier and subclinical inflammatory response that may further increase with laser photocoagulation [4]. By inhibiting cyclooxygenase enzyme, nepafenac and amfenac can inhibit prostaglandin synthesis and reduce such inflammatory response and, hence, minimize macular edema.

Moreover, topical nepafenac can decrease macular edema by a prostaglandin-independent mechanism [13]. Nepafenac decreases VEGF mRNA production in the retina [14] and can suppress VEGF-induced phosphorylation of a downstream molecule [15].

In this prospective study, our primary aim was to evaluate the effect of topical nepafenac eye drops on reducing macular edema and stabilizing visual acuity following laser treatment for diabetic retinopathy. While NSAID have been used systemically for decades, and more recently as topical treatment for various reasons, our study is the first one to evaluate the use of a topical NSAID following laser treatment to minimize macular edema.

Our study showed that BCVA was significantly better in the nepafenac group compared to the control group at all follow-up periods (*P* ≤ 0.01). At 6 months post-PRP, the BCVA was 0.11 ± 0.04 (equivalent to 20/26 Snellen acuity) in the nepafenac group and 0.18 ± 0.08 (equivalent to 20/30 Snellen acuity) in the control group (*P* = 0.007). There was an increase in CFT that was higher in the control group than in the nepafenac group starting from the first month after PRP but became significant only at 4 and 6 months post-PRP. This might suggest that topical NSAIDs should be used for a long time to achieve a beneficial therapeutic effect.

The difference in visual acuity between the two groups in the first two months post-PRP was not associated with significant CFT changes. The worsening of visual acuity was less in the nepafenac group than in the control group starting from 1st month after PRP. It is possible that the early visual acuity changes are related to microstructural changes in the macula and/or an inflammatory process that cannot be detected by the current OCT modalities. This discrepancy between CFT and visual acuity was proven in other studies [16]. However, further studies are needed to confirm such hypothesis.

While topical NSAIDs have been reported to cause corneal epithelial damage, epithelial defects, and even corneal melting, we did not report any significant ocular adverse events during the follow-up. However, as the data about prolonged use of NSAIDs is still scarce, continued monitoring and follow-up of such patients are still needed.

While the study showed a beneficial role of topical nepafenac after laser treatment, it is still unclear from the current study design whether the effect will persist after cessation of treatment. In addition, the study design is unable to determine for how long the treatment should be continued.

One of the limitations of our study is using conventional single-spot argon laser. We tried to overcome the effects of PRP on macular thickness by spacing treatment and using small number of shots per session. Results of our study could vary with newer modalities of multispot and multiwavelength lasers that need to be evaluated in other clinical trials. Although development of macular edema post multispot pattern laser PRP had been proven, the effect of topical nepafenac needs evaluation with that modality of laser [17, 18]. Other studies showed better effects on macular thickness with multispot lasers compared to those with single spot lasers [19–21].

In the current study, patients were randomized, and the observer was masked to the treatment assigned. While sample size calculation was done to ensure that the number of recruited patients was enough, the total number of patients was quite small to allow stratification based on baseline data. However, the difference in the baseline characteristics between both groups was statistically insignificant.

To conclude, topical nepafenac can prevent progressive increase in CFT and can help in stabilizing BCVA after laser treatment for diabetic patients. Further studies are needed to determine for how long treatment should be continued and whether or not there might be a rebound increase in CFT after its discontinuation.

## Figures and Tables

**Table 1:** Baseline characteristics of studied patients.

	Nepafenac group (*n* = 30 eyes)	Control group (*n* = 30 eyes)	*P* value
Age (years) mean ± SD	47.6 ± 6.3	46.4 ± 5.6	0.91
Female sex, *n* (%)	16 (53.3%)	18 (60%)	0.89
Type of diabetes, *n* (%)			
Type I	11 (36.7%)	10 (33.3%)	0.82
Type II	16 (53.3%)	18 (60%)	
Uncertain	3 (10%)	2 (6.7%)	
Duration of diabetes (years) mean ± SD	9.3 ± 2.9	9.5 ± 2.7	0.87
Hypertension, *n* (%)	18 (60%)	17 (56.7%)	1.00
HBA1c (%) mean ± SD	8.1 ± 1.1	8.2 ± 1.2	0.88
Type of diabetic retinopathy			
Severe NPDR	9 eyes (30%)	11 eyes (36.7%)	0.87
PDR	21 eyes (70%)	19 eyes (63.3%)	0.89

NPDR: nonproliferative diabetic retinopathy; PDR: proliferative diabetic retinopathy; SD: standard deviation.

**Table 2:** BCVA and lines of deterioration in both groups.

	Pre-PRP	1 month	2 months	4 months	6 months	Lines lost of BCVA at 6 months
Nepafenac group (logMAR) mean ± SD	0.06 ± 0.06	0.09 ± 0.06	0.09 ± 0.06	0.11 ± 0.05	0.11 ± 0.04	1.0 ± 1.0
Control group (logMAR) mean ± SD	0.07 ± 0.07	0.14 ± 0.08	0.17 ± 0.08	0.19 ± 0.09	0.18 ± 0.08	1.96 ± 1.07
*P* value	0.432	0.011	0.0051	0.0066	0.0072	0.0054

**Table 3:** Central foveal thickness in both groups.

CFT	Baseline	1 month	2 months	4 months	6 months
Nepafenac group (*μ*m) mean ± SD	191.63 ± 8.82	200.44 ± 9.56	205.56 ± 9.44	208.22 ± 9.91	210.85 ± 11.11
Control group (*μ*m) mean ± SD	182.68 ± 9.55	204.18 ± 10.90	210.25 ± 13	219.68 ± 16.42	228 ± 19.79
*P* value	0.091	0.219	0.111	0.003	0.0023

CFT: central foveal thickness.

**Table 4:** Ocular adverse events during follow-up.

Ocular adverse effects	Nepafenac group (*n* = 30 eyes)	Control group (*n* = 30 eyes)
Blurred vision	5 (16.7%)	3 (10%)
Foreign body sensation	2 (6.7%)	1 (3.3%)
Itching	2 (6.7%)	1 (3.3%)
Eye pain	1 (3.3%)	1 (3.3%)
Eye discharge	1 (3.3%)	2 (6.7%)
Chronic redness	3 (10%)	3 (10%)
Conjunctivitis	0	1 (3.3%)
Punctate keratitis	0	0
